# Power-Efficient Beacon Recognition Method Based on Periodic Wake-Up for Industrial Wireless Devices

**DOI:** 10.3390/s18041237

**Published:** 2018-04-17

**Authors:** Soonyong Song, Donghun Lee, Ingook Jang, Jinchul Choi, Youngsung Son

**Affiliations:** IoT Convergence Research Department, Electronics and Telecommunications Research Institute (ETRI), 218 Gajeong-ro, Yuseong-gu, Daejeon 34129, Korea; donghun@etri.re.kr (D.L.); ingook@etri.re.kr (I.J.); spiders22v@etri.re.kr (J.C.); ysson@etri.re.kr (Y.S.)

**Keywords:** low-power, wake-up, beacon recognition, IEEE 802.15.4

## Abstract

Energy harvester-integrated wireless devices are attractive for generating semi-permanent power from wasted energy in industrial environments. The energy-harvesting wireless devices may have difficulty in their communication with access points due to insufficient power supply for beacon recognition during network initialization. In this manuscript, we propose a novel method of beacon recognition based on wake-up control to reduce instantaneous power consumption in the initialization procedure. The proposed method applies a moving window for the periodic wake-up of the wireless devices. For unsynchronized wireless devices, beacons are always located in the same positions within each beacon interval even though the starting offsets are unknown. Using these characteristics, the moving window checks the existence of the beacon associated withspecified resources in a beacon interval, checks again for neighboring resources at the next beacon interval, and so on. This method can reduce instantaneous power and generates a surplus of charging time. Thus, the proposed method alleviates the problems of power insufficiency in the network initialization. The feasibility of the proposed method is evaluated using computer simulations of power shortage in various energy-harvesting conditions.

## 1. Introduction

Recently, wireless devices have been utilized for autonomous monitoring in industrial environments. The wireless devices monitor vibration, sound and temperature data to check the conditions of machinery. These wireless devices communicate with their access points to transmit the sensor data collected. The wireless devices could monitor mobile machines such as vehicles and robots, and their access points may change frequently if the devices are located on vehicular machines. In this case, the initialization procedure for connecting the device to a new access point will increase the power consumption of the wireless devices. The life-time of the wireless devices decreases when the devices are repeatedly attempting a connection. Therefore, power-efficient initialization is an important issue in these environments.

Traditionally, the batteries have been regarded as the primary source of power of the the wireless devices. The researchers in [1,2] considered power conservation approaches. In [1], wireless devices control their sleep time using a fuzzy logic controller. The ratio of throughput to workload and battery level is used as an input of the controller. An inference engine in this controller decides the sleep time of the devices with fuzzy rules. This method enables dynamic adjustment of the power consumption. However, power efficiency in the initial state might be inefficient due to the lack of a priori information such as throughput. In [2], wireless devices control their transmission power on the basis of each location. Beacon nodes transmit a signal with the power level increased step by step until the power reaches the maximum level. Each node calculates the propagation distance, then it selects the appropriate power level. This method leads to initialization delay since it requires time to figure out the optimal power allocation for each device.

These power conservation approaches help to extend the life-time of batteries. However, the batteries still need periodic replacement, requiring man power. The wireless devices are difficult to manage if they are installed in places that are not easy to access. To avoid human intervention, energy harvesters (EHs) are applied as the power source of the wireless devices [3,4]. The EHs generate electrical power as long as their sources of energy are available. Most machines incidentally produce vibrational and thermal energy. In [5], EHs are based on piezoelectric, electrostatic and electromagnetic power generation using mechanical vibration. In [6], thermoelectric EHs generate power using thermal differences. The EHs generate power continuously until the machines halt. However, the amount of generated power is usually at the microwatt level, and this power may be insufficient to operate wireless devices [7,8]. For this reason, the wireless devices should be implemented with low-power consumption.

After rectifying the generated power, super-capacitors store up the direct current (DC)-converted power [9]. In the case of exceeding a circuit-defined threshold, wireless devices are provided with the stored power in the super-capacitors [10]. The wireless devices should communicate with the access points using the stored power only. Since the quantity of industrial data is large, the wireless devices should utilize network bandwidth efficiently [11]. Furthermore, the wireless devices should maintain reliability to protect important data from collisions [12] or in-band interferences [13]. Thus, time-division communication methods are suitable for wireless devices [14]. Upon powering up of the wireless devices, the time-division networks are initialized. Once the wireless devices detect beacons, they start association procedures to allocate guaranteed network resources. However, the wireless devices have no information about the beacon arrival time, and the wireless devices should detect the beacons before power depletion.

In this manuscript, we propose a beacon recognition method based on periodic wake-up for industrial EH wireless devices. For all devices, beacons are located in the same positions in all beacon intervals. For access points, beacons always exist as the head of the beacon interval, and the location of the beacons does not change. For wireless devices, beacons exist at an unknown location in the beacon interval; however, the relative positions of the beacons are the same across all beacon intervals. In order to find the correct beacon location, the proposed method checks for the existence of the beacon using a moving window. The moving window controls the activation time of the receiving mode for the wireless devices. To reduce power consumption, the wireless devices change to the sleep state at the end of the moving window. The moving window shifts its position sequentially in each beacon interval to examine the resources until beacon recognition is finished. For example, the window position for any *i*-th beacon interval is located at the *i*-th resources to check the beacon arrival. Once the beacon is identified, the wireless devices synchronize on the basis of the beacon reception time.

EH wireless devices have to solve the problem of a finite life-time. However, low-power consumption methods should be applied to those kinds of wireless devices due to the insufficient power supply. Until now, many low-power techniques for wireless devices have been proposed; however, these techniques do not consider initialization. The proposed method is one of the low-power consumption techniques related to beacon recognition, and it is effective for network initialization under those power constraint conditions. This proposed method can charge power faster since the required power is less. Beacons are recognized faster, so our proposed method has the merit of fast initialization. Moreover, the proposed method can extend the life-time of battery-supplied wireless devices.

This manuscript provides two contributions. The first is the introduction of the reduction ratio to anticipate the energy efficiency. Engineers could utilize this ratio to design a power supply circuit or control the power consumption of the device. The second is the consideration of the problem of real-world clock drift. This analysis reflects physical layer parameters and provides an example of the applicable beacon interval (BI) range based on the tolerance of the 2.4-GHz specification of the IEEE 802.15.4 standard.

## 2. Related Work

### 2.1. IEEE 802.15.4 Beacon-Enabled Networks

Industrial wireless devices are applicable for monitoring various modules in industrial plant environments [11,12,13,14]. The wireless devices are installed in a wide-ranging area, so they should be managed with low cost and low maintenance. Furthermore, the wireless devices should ensure the reliability of data to figure out the states of machines correctly. The specifications of the IEEE 802.15.4 standard meet the requirements of low power consumption [15]. IEEE 802.15.4 devices can be implemented at a low cost because the physical components for signal processing use a small amount of hardware resources. Even if it is difficult to communicate in target environments such as those surrounded by metallic machinery, IEEE 802.15.4 devices have good reception performance. The signal of the standard is robust to low signal-to-noise conditions because it applies the sequence spread spectrum directly. The radio frequency (RF) sensitivity of the signal is lower than −85 dBm, so receivers secure a sufficient fade margin via a wide dynamic range [16]. Moreover, the IEEE 802.15.4 standard supports a beacon-enabled network, so wireless devices avoid collisions by allocating guaranteed resources. Thus, IEEE 802.15.4 devices are reliable enough, and they are applicable to the target environments.

**Figure 1:**
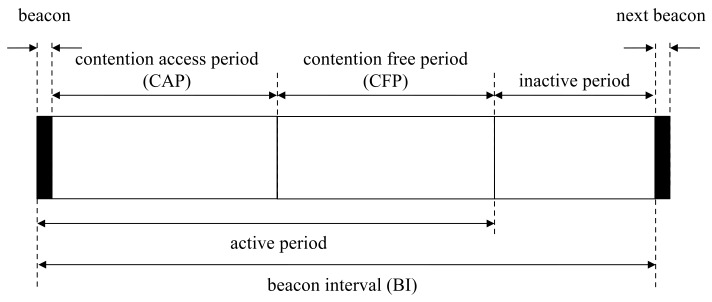
Superframe structure in beacon-enabled networks.

Access points communicate with wireless devices based on the superframe structure in beacon-enabled networks [17]. Access points in beacon-enabled networks manage slots defined by partitioned time logically. The beacon interval in this network is defined by the summation of the active and inactive period, as shown in Figure 1. In the inactive period, all devices belonging to the same network are powered down to save power. The active period is composed of the beacon, the contention-access period (CAP) and the contention-free period (CFP). Beacons transmit by access points to report network information and synchronize devices. In the CAP, devices attempt to connect to access points by random access. In the CFP, devices connect to access points with guaranteed resources by the time-division access in the clear channel. The devices try to associate with access points for addressing. After finalizing the association procedure, the devices are eligible to communicate with access points. Sleep mode is easy to configure for CFP, since the devices only need to continue to receive the mode during the allocated period from the access points [18].

**Figure 2:**
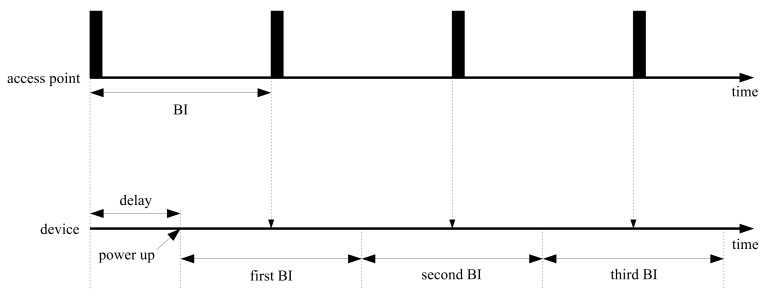
Periodic beacon arrival with random delay.

In Figure 2, the operation flows for access points and devices are represented along the time axis. Access points transmit beacons at a constant rate at the beginning of beacon-enabled networks. Devices start connecting to the access points randomly; however, the devices should control wireless functions to be able to catch incoming beacons. When power supply is sufficient, devices can retain the receive states of successful beacon recognition without termination. However, in the case of a lack of stored power, devices are turned on and off randomly. In the worst case, the devices are powered down repeatedly before beacon arrivals. Power insufficiency may occur by modifying beacon periods; however, the capacity of the power supply circuit is impossible to modify even by changing the network properties.

### 2.2. Medium Access Control Protocols for Low-Power Consumption

Some medium access control (MAC) protocols were proposed related to periodic wake-up schemes for EHs. In [19], on-demand MAC (ODMAC) is a representative energy-efficient protocol. A device using this protocol broadcasts status information via small-length packets to neighboring devices. This method introduces an opportunistic forwarding scheme to avoid energy waste. To determine the duty cycle, this method monitors the amount of generated power using the battery level and a threshold. In [20], an energy-harvested receiver-initiated MAC (ERI-MAC) concatenates small packets without packet headers, and it eliminates overhead information. Furthermore, this method regards acknowledgment packets as the beacon packets. These two key ideas contribute to enhancing the energy efficiency; however, they make assumptions to be able to know the harvesting rate. In [21], RF-MAC supports both communication and wireless charge in the same frequency. This method perfectly overcomes the limitations of battery-powered systems. In this environment, devices are not able to communicate during the charging period; therefore, throughput is not optimized. Furthermore, in this approach, the distance of each device from an access point affects the charging performance. Devices located in the outer boundary may lose the communication opportunity due to the lack of power.

Receiver-initiated protocols begin network initialization by transmitting beacons from one of the devices. These protocols require additional procedures to synchronize neighboring devices [22,23]. In beacon-enabled networks, however, all devices should synchronize with the access points. The access points control the network resources. Each device should first figure out the properties of the resources from beacons and make an attempt to access them. This initialization procedure should support the low power consumption specification. In order to find beacons transmitting from access points, a modified duty-cycle approach is needed.

## 3. Periodic Wake-Up for Beacon Recognition

In this manuscript, a periodic wake-up method is considered to reduce wasted power. This proposed method uses the characteristics of periodic beacon transmission, and a moving window controls devices to carry out receive and sleep mode interchangeably. The moving window is designed to cover beacon arrivals by sliding as much as a BI until the correct beacon is detected. The proposed method is focused on beacon recognition without the synchronization of neighboring devices. We define some variables to design the moving window for beacon inspection. We assume that $N_{BI}$ is the number of BIs for observation, $T_{BI}$ is the time for BI, P(i) is the *i*-th BI, *i* is an integer in the range of zero to $N_{BI}-1$ and $t_{O}$ is the offsets between the initial and actual BI from the perspective of the devices.

### 3.1. Moving Window Design

**Figure 3:**
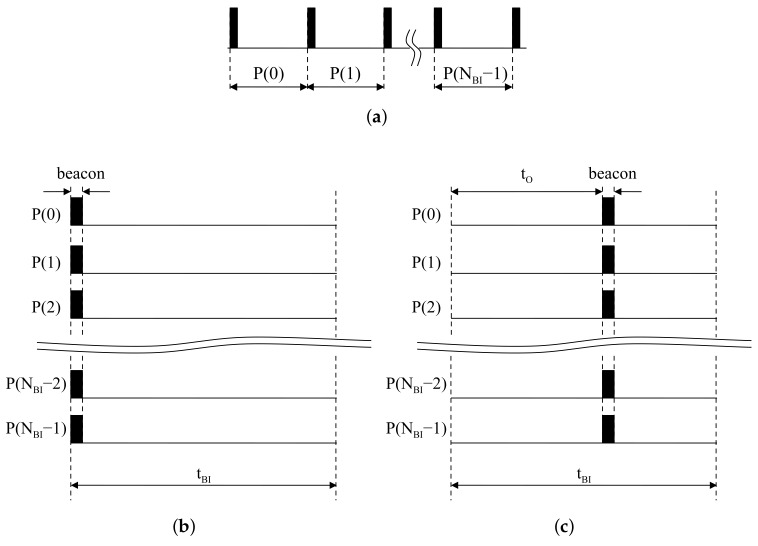
Beacon alignment: (**a**) Periodic beacons at an access point; (**b**) Piled beacons at an access point; (**c**) Piled beacons at a device.

Once network configuration by access points is finished, BIs are rarely changed since the transmission rate of beacons is constant. In terms of access points, beacons always exist at the first slot in all BIs. Continuous BIs with respect to access points are represented in Figure 3a. Piling the sliced BIs, beacons are always located at the front of all BIs, as shown in Figure 3b. In the same situation, a device starting after $t_{O}$ is assumed to listen to the the wireless channel to catch signals from access points. The offset $t_{O}$ is regarded as the time difference between the beginning of the device and the first beacon reception. The partitioning result of the listening time into $T_{BI}$ is represented in Figure 3c. The device is started with delay as $t_{O}$ at the first BI. The time difference between the beginning of the second BI and the second incoming beacon also corresponds with $t_{O}$. Piling the partitioned results, beacons in the device appear at the same location inside BIs.

**Figure 4:**
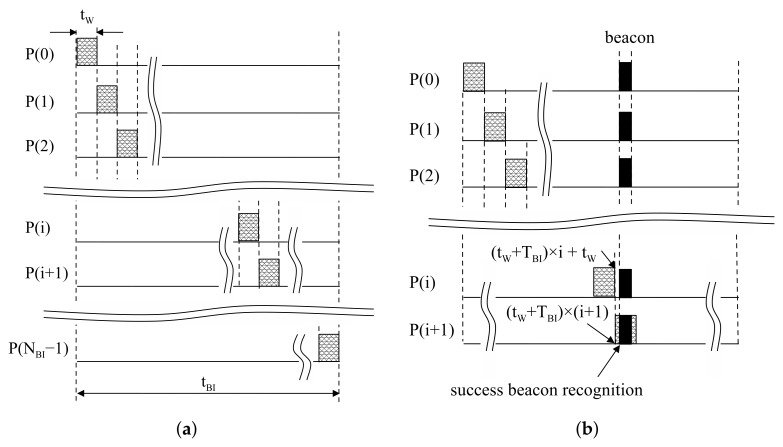
Operation of a proposed moving window: (**a**) Design of the proposed moving window; (**b**) An example of success in beacon recognition.

The proposed moving window detects aligned beacons at the same position in each BI. The moving window is designed to check the reception of a beacon for resources at each BI. In this method, the receive mode is activated during $t_{W}$ in $T_{BI}$. At the next $T_{BI}$, the remaining resources are checked in the same way. This process runs repeatedly until beacon reception is successfully finished, as shown in Figure 4b. Beacon recognition is able to finish within $N_{BI}\times T_{BI}$ by the proposed moving window.

The size of the moving window is defined as $T_{BI}/N_{BI}$ to recognize beacons within $N_{BI}$ trials. Then, devices stay in sleep mode, except the activated time for the moving window. At P(i) in Figure 4a, the moving window is located at $\left(t_{W}+T_{BI}\right)\times i\leq t<t_{W}+\left(t_{W}+T_{BI}\right)\times i$. Similarly, at P(i+1) in Figure 4a, the moving window is located at $\left(t_{W}+T_{BI}\right)\times \left(i+1\right)\leq t<t_{W}+\left(t_{W}+T_{BI}\right)\times \left(i+1\right)$. The tail of the *i*-th moving window is $t_{W}+\left(t_{W}+T_{BI}\right)\times i$, and the head of the i+1-th moving window is $\left(t_{W}+T_{BI}\right)\times \left(i+1\right)$; then, $\left(t_{W}+T_{BI}\right)\times \left(i+1\right)-\left\{t_{W}+\left(t_{W}+T_{BI}\right)\times i\right\}=T_{BI}$. Thus, the proposed moving window can be realized by staying in sleep mode during $T_{BI}$ after receiving during $t_{W}$. A period of the moving window is $t_{W}+T_{BI}$, so the duty cycle is calculated with $t_{W}/\left(T_{BI}+t_{W}\right)$. From those results, the switching interval of the device can be generalized as:

(1) $$\begin{array}{ccc} t_{ON}\left(i\right) & = & \left(T_{BI}+t_{W}\right)\times i \end{array}$$

(2) $$\begin{array}{ccc} t_{OFF}\left(i\right) & = & \left(T_{BI}+t_{W}\right)\times i+t_{W} \end{array}$$

where $t_{ON}\left(i\right)$ is the starting time of receive mode in P(i) and $t_{OFF}\left(i\right)$ is the starting time of sleep mode in P(i).

### 3.2. Calculation of Power Consumption

In this subsection, the power consumption of the proposed method is calculated. In Table 1, the parameters related to this calculation are described. The amount of the normalized power consumption is calculated by the total power consumption divided by the summation of the receiving delay.

As shown in Figure 3b, for a device, the time offset is not changed in BI. We assume $t_{O,N_{BI}>1}=t_{q}+t_{r}$ to represent $t_{O,N_{BI}>1}$ in terms of $t_{W}$, and $t_{O,N_{BI}>1}=t_{O,N_{BI}=1}$. The $t_{q}$ is the verified time for the absence of a beacon by moving windows, so it is a multiple of $t_{W}$. The $t_{r}$ is the time for the moving window at beacon reception. It is the remainder of $t_{O,N_{BI}=1}$ divided by $t_{W}$, so it is always less than $t_{W}$. If the beacon is detected at the (i+1)-th BI, the index of BIs *i* can be written as:

(3) $$\begin{array}{c} i=\left\lfloor \frac{t_{O,N_{BI}=1}}{t_{W}}\right\rfloor \end{array}$$

$t_{q}$ and $t_{r}$ are:

(4) $$\begin{array}{ccc} t_{q} & = & i\times T_{BI} \end{array}$$

(5) $$\begin{array}{ccc} t_{r} & = & \frac{t_{O,N_{BI}=1}}{t_{W}}-i\times t_{W} \end{array}$$

Then, $t_{O,N_{BI}>1}$ can be written as:

(6) $$\begin{array}{ccc} t_{O,N_{BI}>1} & = & i\times T_{BI}+\frac{t_{O,N_{BI}=1}}{t_{W}}-i\times t_{W}\\ & = & \frac{t_{O,N_{BI}=1}}{t_{W}}+i\times \left\{T_{BI}-t_{W}\right\} \end{array}$$

Therefore, the total power consumption can be written as:

(7) $$\begin{array}{ccc} P_{T,N_{BI}=1} & = & P_{RX}\times t_{O,N_{BI}=1}\\ P_{T,N_{BI}>1} & = & \left\{P_{RX}\times t_{W}+P_{SLEEP}\times \left(T_{BI}-t_{W}\right)\right\}\times i+P_{RX}\times \left\{\frac{t_{O,N_{BI}=1}}{t_{W}}-i\times t_{W}\right\} \end{array}$$

(8) $$\begin{array}{cc} = & P_{RX}\times \frac{t_{O,N_{BI}=1}}{t_{W}}+P_{SLEEP}\times \left(T_{BI}-t_{W}\right)\times i \end{array}$$

For $N_{BI}=1$, the instantaneous power in a single BI is the same as the total power consumption, so $P_{N,N_{BI}=1}=P_{T,N_{BI}=1}$. In the case of $N_{BI}>1$, the instantaneous power in the same period can be written as $P_{N,N_{BI}>1}=P_{T,N_{BI}>1}/\left(i+1\right)$. Then, a reduction ratio for the power consumption can be calculated by:

(9) $$\begin{array}{ccc} \frac{P_{N,N_{BI}>1}}{P_{N,N_{BI}=1}} & = & \frac{1}{i+1}\times \frac{P_{T,N_{BI}>1}}{P_{T,N_{BI}=1}}\\ & = & \frac{P_{RX}\times t_{O,N_{BI}=1}/t_{W}+P_{SLEEP}\times \left(T_{BI}-t_{W}\right)\times i}{\left(i+1\right)\times P_{RX}\times t_{O,N_{BI}=1}}\\ & = & \frac{1}{\left(i+1\right)\times t_{W}}+\frac{P_{SLEEP}}{P_{RX}}\times \frac{T_{BI}-t_{W}}{t_{O,N_{BI}=1}}\times \frac{i}{i+1} \end{array}$$

In the case of $N_{BI}>1$, $\left(i+1\right)\times t_{W}>1$. Usually $P_{RX}\gg P_{SLEEP}$, so the reduction ratio is definitely smaller than one. This result shows that the instantaneous power can be reduced by the proposed method.

Next, let us consider devices’ operation. The devices are operable when the generated power is greater than the power consumption. From this fact, the conditions are derived as follows.

(10) $$\begin{array}{ccc} P_{T,N_{BI}=1} & < & P_{EH}\times t_{O,N_{BI}=1} \end{array}$$

(11) $$\begin{array}{ccc} P_{T,N_{BI}>1} & < & P_{EH}\times t_{O,N_{BI}>1} \end{array}$$

Beacon detection for $N_{BI}=1$ needs time for power generation above at least $P_{T,N_{BI}=1}$. At the initial state, devices based on the conventional method may be operated along with a delayed start. Devices should wait for the power to be sufficiently charged. In the case of $N_{BI}>1$, the generated power $P_{EH}\times t_{O,N_{BI}>1}$ will be greater than $P_{EH}\times t_{O,N_{BI}=1}$ since $t_{O,N_{BI}>1}>t_{O,N_{BI}=1}$. This means that devices initialized by the proposed method may reach operable power faster than the conventional method. Therefore, the delay of the proposed method can be shorter than the conventional method.

### 3.3. Consideration of Clock Drift

In the standard of the IEEE 802.15.4 [15], the tolerance is specified as ±40 ppm. Therefore, one second of the devices could be perceived in the range of 0.99996–1.00004 s. Considering the transmitter and receiver, the maximum clock drift could be up to 80 ppm. We assume a beacon is supposed to arrive at the *i*-th window in the case of no clock drift and perfect link-layer communication conditions. Then, we consider two difficult situations by the real-world clock drift with respect to the devices.

The first situation assumes that a device runs fast with the maximum clock drift. Beacons in the device are moving in the direction toward the tail of beacon intervals gradually. Suppose that an (i−1)-th beacon is located at the tail of an *i*-th window. At the next step, the beacon might deviate from the *i*-th window as 0.008% of a beacon interval. In this case, the *i*-th window should be extended as much as 0.008% at the last step of the beacon recognition. This extension probably will not affect most of situations due to the lack of sufficient power supply. This could be simply realized by turning off the radio after finishing the beacon recognition procedure.

The second situation assumes that a device runs slow with the maximum clock drift. Beacons in the device are moving in the direction toward the head of beacon intervals gradually. Suppose that an (i−1)-th beacon is located at the head of an *i*-th window. This is a similar situation as shown above; however, a part of the beacon packet cannot be used for reception. This situation could lead to serious performance degradation due to packet loss. An actual packet is composed of a PHY (physical) header and a PHY payload. Here, a preamble is located at the beginning of the PHY header. In the preamble, eight identical symbols are allocated, and they utilize a coarse timing synchronization. This synchronization is the first step to detect packets with symbol level accuracy. Usually, the coarse timing can be calculated by autocorrelation based on the moving average with two identical symbols. This means that the remaining six symbols are redundant parts of the packet. For example, the symbol time is 16 μs at a 2.4-GHz O-QPSK specification. Therefore, the packet has a 96 μs margin. The beacon interval can be calculated by Equation (12). At *BO* = 7, the beacon interval is 0.98304 s, and the maximum clock drift is around 78.6 μs. Based on this analysis, our method can be used under the condition *BO* < 8 without any modification.

In the reference standard, 15 slots are available at the same superframe since the first slot is only available for beacon packets. This means only 15 devices can communicate simultaneously. Furthermore, the maximal packet length is 133 bytes including PHY overheads. A signal of one byte is represented during 32 μs. Therefore, the maximal packet time is 4.256 ms. If *BO* = 7, the maximum slot duration is 7.68 ms. If reliability is important, the packet length should be shorter since long packets increase the probability of errors occurring. In this standard, link-layer performance is guaranteed to be lower than 1% packet error probability at −85 dBm RF sensitivity with a 20-byte packet. Considering bandwidth efficiency and reliability, the beacon interval and packet length should not be too long in an industrial wireless network. For this reason, the condition of *BO* < 8 is sufficient for the practical target environment.

## 4. Evaluation of the Proposed Method

In this section, we prove the outstanding performances of the proposed method in terms of power consumption and recognition time by computer simulations. For the simulation scenario, each wireless device is directly connected to an access point. The performance results are represented in terms of power and delay with various simulation parameters. In this simulation, the proposed method is compared to a straightforward method. We assume that the straightforward method holds the receive mode until a device detects a beacon. The power supply in the straightforward method should secure power in order for the receive mode to be operable for at least one BI before starting the device. The results for $N_{BI}=1$ show the performance of the straightforward approach since a BI is not partitioned; whereas, the results for the other conditions show the performance of the proposed method.

### 4.1. Simulation Setup

QualNet [24] is an event-driven communication system simulation software. This software supports fundamental functions for the IEEE 802.15.4 standard [25]. Using this library, the proposed method is implemented by modifying a part of the physical and MAC layer. In the physical layer, wireless signals are automatically calculated by reflecting radio propagation. At the transmitter, the radio properties are configured in relation to the transmission power. In wireless channels, path losses are calculated using the distance between wireless devices. At the receiver, incoming packets are verified as to whether error has occurred based on RF sensitivity and the signal-to-noise ratio. In the MAC layer, some commands are exchanged for device control. MAC functions exchange request and response messages to control remote devices. Some MAC messages should handle related events before the timer has expired.

**Figure 5:**
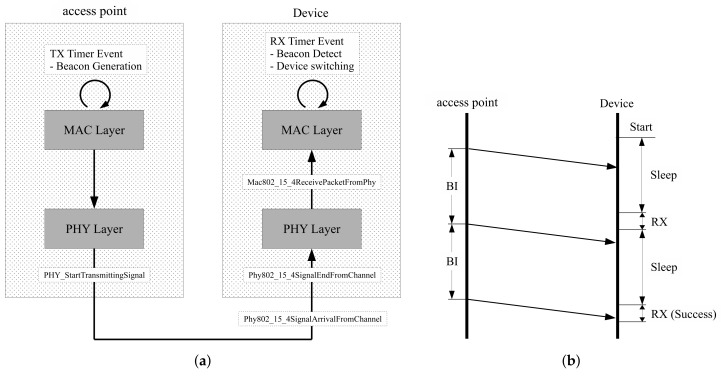
Configuration of simulation software: (**a**) Beacon handling process; (**b**) Example of operation flow.

Simulations are focused on the implementation of the beacon life-cycle, as shown in Figure 5a. Access points generate periodic beacons using the timer function. Beacon packets are transmitted when the timer has expired. In the physical layer, beacon packets are moved to a software kernel through the PHY StartTransmittingSignal function. The kernel handles the propagation delay, then transfers beacon signals to devices. The physical layer of the devices handles received signals with interface functions such as Phy802 15 4SignalArrivalFromChannel and Phy802 15 4SignalEndFromChannel. These functions decide whether the received signals are available by calculating the packet error probability based on the path loss information. Then, successful signals move to the next step through Mac802 15 4ReceivePacketFromPhy. In this procedure, the devices check the remaining energy. Packets move to the next step if the accumulated energy is sufficient to receive. The proposed method is implemented using MAC layer interfaces since the MAC layer is able to control device conditions. The proposed moving window is realized using timers that change the receive and sleep state by turning onwhen the device timers have expired.

In this simulation, the propose method is evaluated using the MICAz model [26]. The MICAz model is one of the IEEE 802.15.4 device models that is provided by the simulation software. MICAz is powered by two AA batteries, and its radio is equipped with a CC2420 transceiver [27]. This model consumes a current of 18.8 mA in receive mode. while 0.02 mA in sleep mode. The AA batteries output 1.5 V per piece, so the input voltage of the radio is 3 V. Power is a result of multiplying the voltage and current; therefore, the power is calculated as 56.4 mW in receive mode and 0.06 mW in sleep mode, respectively. To monitor the power consumption in devices, we observe the change of energy stored in the super-capacitor. We assume that a power supplier has perfect energy efficiency. Then, the super-capacitor stores up the difference between generated and consumed energy. When the device is in sleep mode during $t_{1}$ and in receive mode during $t_{2}$, the energy $E_{CAP}$ stored in the super-capacitor during $t_{1}+t_{2}$ is calculated as $E_{CAP}=\left\{P_{EH}-\left(P_{SLEEP}+P_{RX}\right)\right\}\times \left(t_{1}+t_{2}\right)$. Once the beacon has been received, the device will restart randomly within a BI.

**Figure 6:**
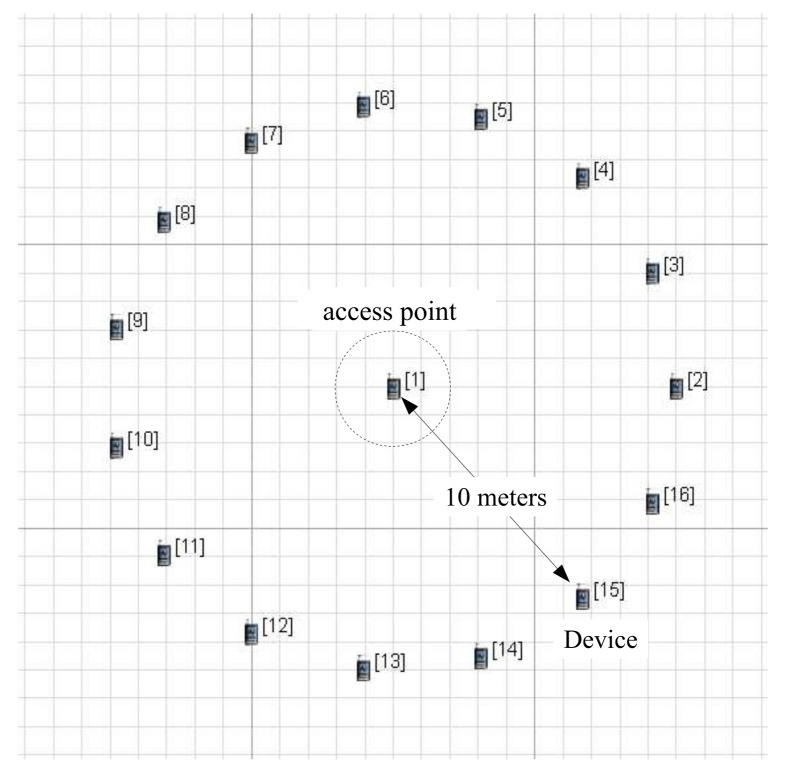
Deployment of an access point and devices in scenario plane.

As shown in Figure 6, each device is 10 meters away from an access point in the scenario. This configuration does not cause link-layer errors, so the performance results depend on the simulation conditions alone. The beacon order (BO) decides the length of a BI, and it is configured as 3. The BI is related to the BO as follows:

(12) $$BI=aBaseSuperframeDuration\times 2^{BO}$$

where aBaseSuperframeDuration is the symbol time for a superframe, defined in the IEEE 802.15.4 standard, and set to a constant number: 960. The rest of the simulation parameters are described in Table 2.

### 4.2. Simulation Results

**Figure 7:**
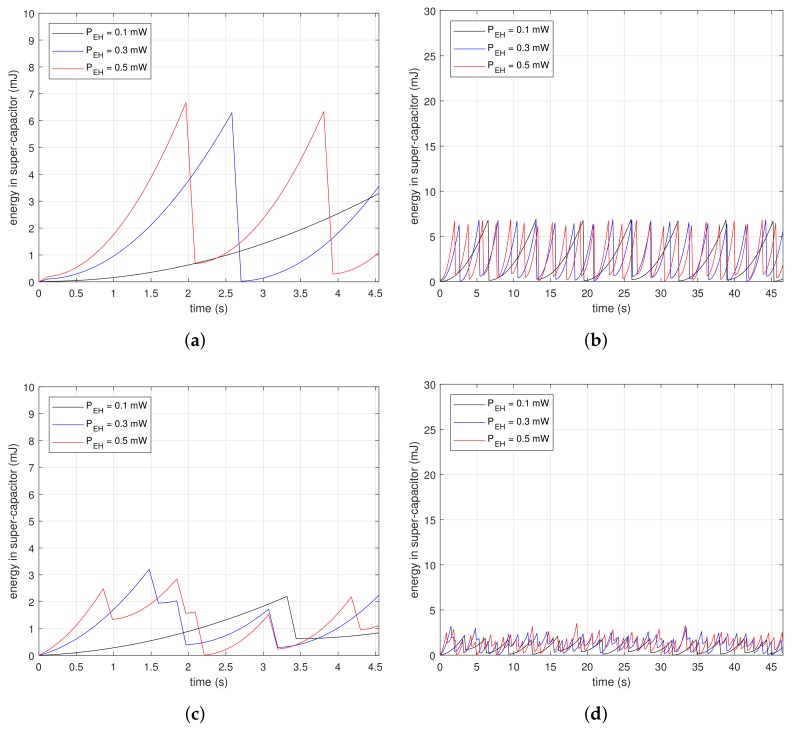

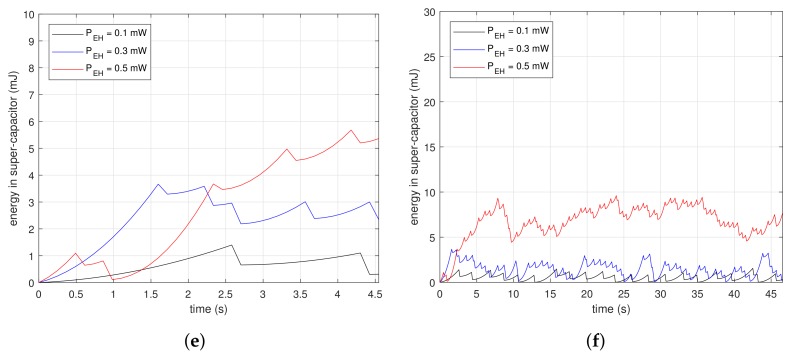
Initial amount of stored energy in a super-capacitor in conditions of $N_{BI}=1$, $N_{BI}=4$, and $N_{BI}=8$: (**a**) From 0 to 4.5 s for $N_{BI}=1$; (**b**) From 0 to 47 s for $N_{BI}=1$; (**c**) From 0 to 4.5 s for $N_{BI}=4$; (**d**) From 0 to 47 s for $N_{BI}=4$; (**e**) From 0 to 4.5 s for $N_{BI}=8$; (**f**) From 0 to 47 s for $N_{BI}=8$.

Figure 7 shows measurements of the energy changes in a super-capacitor at the beginning of beacon recognition. The subfigures are the results of drawing the energy variation along time in the condition of $N_{BI}=1$, $N_{BI}=4$ and $N_{BI}=8$. Figure 7a,c,e are the magnified results of Figure 7b,d,f, respectively. The saw-shape graphs imply the energy property, which is either charging or consumption. Devices accumulate energy in the super-capacitor when the slopes are ascending, while they drain the energy out of the super-capacitor when the slopes are descending dramatically. Figure 7a,b are the results for $N_{BI}=1$. In Figure 7a, vertical slopes appear periodically at each $P_{EH}$. At the inflection points, devices consume energy instantaneously, as much as the descending depth. The first inflection points for $P_{EH}=0.1$ mW and $P_{EH}=0.5$ mW are 4.2 and 1.9 s, respectively. The reception delays are inversely proportional to $P_{EH}$ since the higher $P_{EH}$ finishes charging faster. In Figure 7b, the charge–discharge process appears repeatedly. Figure 7c,d are the results for $N_{BI}=4$. In Figure 7c, the first inflection point is reached faster than as shown in Figure 7a. Devices with the proposed method only need to charge as much as the time of the moving windows; therefore, they finish collecting the necessary energy faster than the case where $N_{BI}=1$. Furthermore, the descending depth of the slopes is less than in the case where $N_{BI}=1$. This means the proposed method consumes less energy for beacon recognition. The graphs in Figure 7d are at a lower position than in Figure 7b. These results show that the proposed method is suitable for low power devices. Figure 7e,f are the results for $N_{BI}=8$. In Figure 7e, the first inflection point is reached faster than shown in Figure 7c, and also the descending depth is less. Moreover, the stored power can grow as shown in Figure 7f due to the low power property of the proposed method.

**Figure 8:**
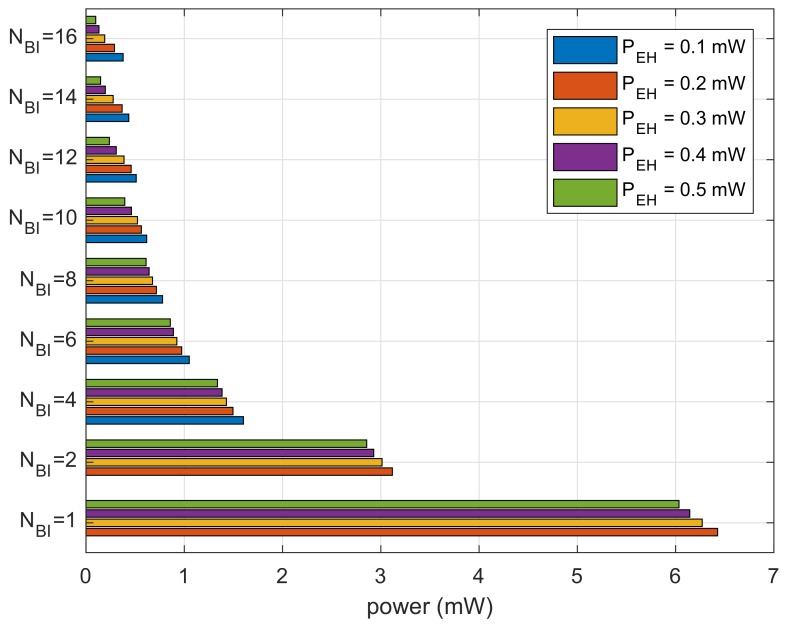
Average power consumption to recognize one beacon.

Figure 8 shows the results for cumulative power spent divided by the number of recognized beacons. The results show the average power consumption for receiving a beacon. We can observe that the bar graphs are decaying inversely proportional to $N_{BI}$ because devices with a smaller size of the moving window expend less power. For the same $N_{BI}$, the lower $P_{EH}$ expends more power. At a lower $P_{EH}$, the devices expend power in sleep mode during necessary power charging.

**Figure 9:**
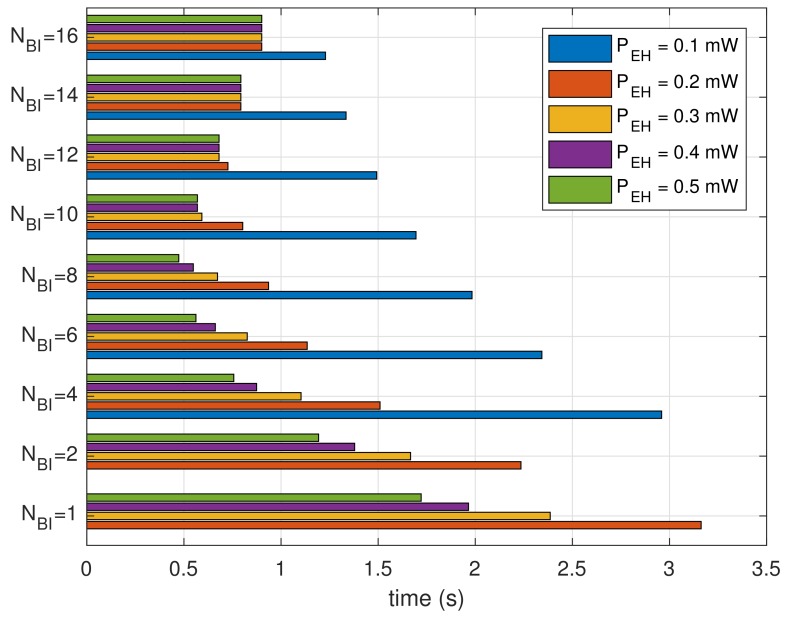
Average time spent to recognize one beacon.

Figure 9 shows the results of dividing the cumulative reception delay by the number of recognized beacons. The results show the average time spent for receiving a beacon. For each generation amount, the bar graphs show a concave shape along the given $N_{BI}$. The beacon recognition delay is minimized at $N_{BI}=8$ for $P_{EH}=0.5$ mW. For $N_{BI}<8$, the beacon recognition delay decreases by reducing the power consumption due to shortening of the moving window. For $N_{BI}>8$, on the other hand, the results of beacon recognition delay increase because the moving window spends more time traveling to search for the beacons.

**Figure 10:**
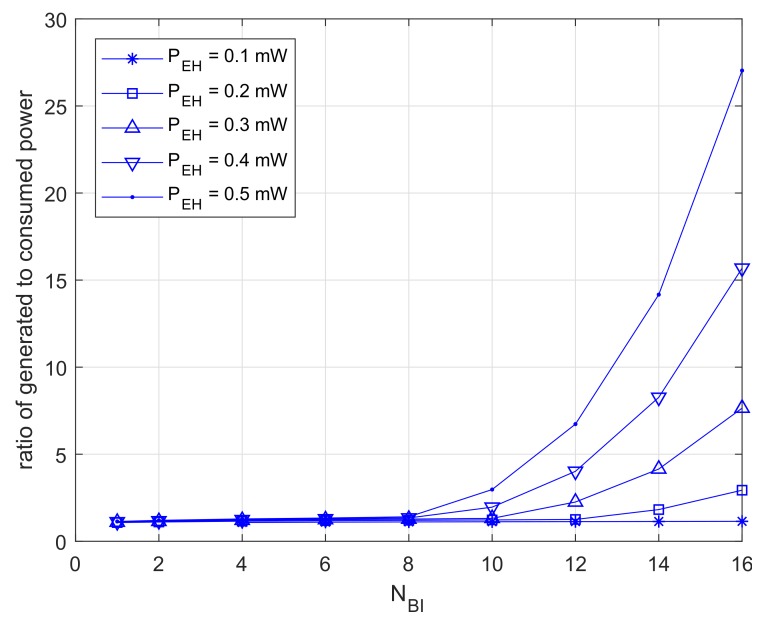
Ratio of excessive power.

Figure 10 shows the results for dividing the generation quantity by the power usage. The results are close to one at $N_{BI}\leq 8$, and this means that the devices consume most of the power stored. Furthermore, those results are increasing exponentially at $N_{BI}>8$. In this case, devices are able to retain excess power.

**Figure 11:**
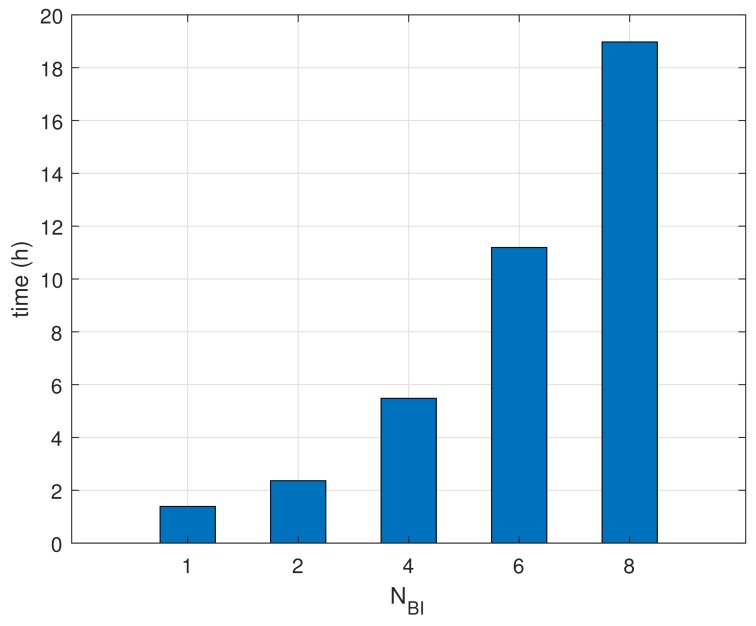
Comparisons of life-time.

Finally, Figure 11 shows the measurements of the life-time in the condition of devices using batteries. The life-time for $N_{BI}=1$ is reached at the very beginning since the devices consume power continuously until finishing beacon recognition. The life-time patterns are growing proportional to $N_{BI}$. These results show that the proposed method helps extend the life-time of battery-based wireless devices.

## 5. Discussion and Conclusions

This manuscript proposed a power-efficient beacon recognition method based on periodic wake-up in network initialization for wireless devices. In beacon-enabled communication between access points and devices, the proposed method reduced the power consumption for beacon detection. A moving window approach was introduced that was designed for wake-up scheduling, and it made it possible to find unknown beacon arrival times. The moving window examined the existence of beacons at partial resources in each BI. After changing the position of the moving window shifted to the next resource, the proposed method checked for the beacons’ existence again. Wireless devices went to sleep mode to save power, except when the moving window was in process. Therefore, our method provided power stability for wireless devices by consuming low power and securing the charging time.

The proposed method enables the reduction of instantaneous power consumption by spreading out the required energy in the time domain. This method reduces the charging time to reach the necessary energy level, so it could finalize beacon recognition procedure faster compared to the straightforward method. Additionally, this approach utilizes generated power efficiently by putting the right power at the right time; whereas, the proposed method leads to unnecessary delay in the condition of holding sufficient energy. Some networks using a long beacon period could result in packet loss due to clock drift. Thus, the proposed method should be applied carefully to low-speed communication systems or massive networks.

The proposed method is suitable for frequently-initialized and power-constrained wireless devices. Furthermore, this method is applicable to wireless devices because it facilitates recollecting wasted energy in industrial plants. Consequently, our method contributes to reducing the maintenance cost of industrial wireless devices based on a beacon-enabled network. Industrial wireless devices for plant monitoring relied on the previously-mentioned communication conditions. The wireless devices on relocatable machinery change access points frequently in these environments. The initialization of beacon-enabled networks can succeed under insufficient power supply since our method provides power-efficient beacon recognition. With the help of computer simulations, our results proved effective in the target environments.

## Figures and Tables

**Table 1:** Description of variables related to beacon detection procedure.

Variables	Description
$t_{O,N_{BI}=1}$	time offsets to finishing beacon detection for $N_{BI}=1$
$t_{O,N_{BI}>1}$	time offsets to finishing beacon detection for $N_{BI}>1$
$P_{N,N_{BI}=1}$	power for $N_{BI}=1$ in a BI
$P_{N,N_{BI}>1}$	power for $N_{BI}>1$ in a BI
$P_{T,N_{BI}=1}$	total power for $N_{BI}=1$
$P_{T,N_{BI}>1}$	total power for $N_{BI}>1$
$P_{SLEEP}$	power consumption in sleep mode
$P_{RX}$	power consumption in receive mode
$P_{EH}$	generated power

**Table 2:** Configuration of simulation parameters.

Parameters	Value
simulation time (s)	86,400
number of devices	15
radio tx power (dBm)	0
device model	MICAz
battery capacity (mWh)	30
BO	3
$N_{BI}$	1, 2, 4, 6, 8, 10, 12, 14, 16
$P_{EH}$ (mW)	0.1, 0.2, 0.3, 0.4, 0.5

## Abbreviations

BI	Beacon interval
BO	Beacon order
CAP	Contention access period
CFP	Contention free period
DC	Direct current
dBm	Decibel-milliwatts
EH	Energy harvester
GHz	Gigahertz
MAC	Medium access control
mA	Milli-Ampere
mW	Milli-Watt
O-QPSK	offset quadrature phase shift keying
PHY	Physical
ppm	Parts-per million
RF	Radio-frequency
SFD	Start-of-frame delimiter
